# Association between inflammatory burden index combined with D-dimer and acute kidney injury in elderly patients

**DOI:** 10.3389/fmed.2026.1764718

**Published:** 2026-04-28

**Authors:** Shuqi Liu, Xingyang Zhao, Shuwang Ge, Ningxu Li

**Affiliations:** 1 Division of Nephrology, Liyuan Hospital, Tongji Medical College, Huazhong University of Science and Technology, Wuhan, China; 2 Division of Nephrology, Tongji Hospital, Tongji Medical College, Huazhong University of Science and Technology, Wuhan, China

**Keywords:** acute kidney injury, coagulation and fibrinolysis, D-dimer, elderly patients, inflammatory burden index, inflammatory immune response

## Abstract

**Background:**

The interplay between inflammation and coagulation is central to the pathophysiology of acute kidney injury (AKI). This study aimed to explore the association between IBI and the incidence of AKI in the elderly, as well as the intricate connections between IBI, D-dimer, and AKI.

**Methods:**

The data came from the clinical records of patients aged ≥ 65 years hospitalized at Wuhan Tongji Hospital between January 2018 and December 2022. We evaluated the predictive efficacy of IBI and other inflammatory indicators for AKI by receiver operating characteristic (ROC) analysis. Restricted cubic spline (RCS) and Logistic regression models were employed to examine the correlation between IBI, D-dimer, and AKI risk. Furthermore, ROC analysis, integrated discrimination improvement (IDI), and net reclassification improvement (NRI) were performed to assess the predictive value of the combined IBI-D-dimer index. The bidirectional mediating effects of IBI and D-dimer in AKI were validated using the bootstrap method.

**Results:**

This study involved 69,738 participants. The median age was 71.24 (67.75, 76.46) years, and 25,831 (37.0%) were female. During hospitalization, 3,755 (5.4%) elderly patients developed AKI. ROC analysis demonstrated that, compared to conventional inflammatory indicators, Log IBI had superior predictive value for AKI in elderly patients (AUC = 0.719, 95% CI 0.711–0.727, *p* < 0.001). In multivariate analyses, the highest quartile of Log IBI (OR = 2.526, 95% CI 2.193–2.918, *p* < 0.001) and D-dimer levels (OR = 2.493, 95% CI 2.184–2.854, *p* < 0.001) were independently linked to an elevated risk of AKI. A significant multiplicative interaction between Log IBI and D-dimer was observed (*p*-interaction < 0.001). The combined index further improved risk reclassification, with participants exhibiting concurrent elevations of both markers conferring the highest AKI risk (OR = 2.640, 95% CI 2.328–3.002, *p* < 0.001). Mediation analysis revealed bidirectional effects: D-dimer mediated 31.30% (*p* < 0.001) of Log IBI’s association with AKI, while Log IBI mediated 6.18% (*p* < 0.001) of D-dimer’s effect.

**Conclusion:**

Concurrent elevation of IBI and D-dimer was associated with the greatest risk of elderly AKI. The dynamic monitoring of IBI and D-dimer may help identify high-risk elderly patients for AKI.

## 1 Introduction

Acute kidney injury (AKI) is a clinical syndrome precipitated by multiple etiological factors such as ischemia, infection, and obstruction, and it is marked by a sharp drop in renal function (1). The incidence of AKI has shown a steady annual increase in recent years. A meta-analysis indicated that the global incidence of AKI in adults reaches 22%, with up to 50% in intensive care unit (ICU) patients, and its duration and disease progression are highly correlated with mortality, imposing a substantial medical burden (2). Notably, elderly patients have become a high-risk group for AKI due to the increased prevalence of underlying diseases such as hypertension, diabetes, and coronary artery disease (CAD), as well as age-related changes in renal physiology (3). Therefore, early detection of modifiable risk factors and high-risk populations is essential for adopting targeted preventive measures to lower the incidence of AKI in the elderly.

Excessive activation of the inflammatory responses represents a central mechanism in the pathogenesis of AKI, with multiple immune cells and pro-inflammatory cytokines collectively promoting glomerular and tubular cell injury (4). Early studies have identified that C-reactive protein (CRP) and neutrophil-to-lymphocyte ratio (NLR) are independent predictors for the onset and prognosis of AKI (5, 6). By integrating CRP and NLR, the novel biomarker Inflammatory Burden Index (IBI) reflects the dynamic crosstalk between inflammation and immune responses, showing great clinical applicability. IBI was initially developed to predict the prognosis of cancer patients and was first proposed by Xie et al. in 2022 as a marker to quantify systemic inflammation levels (7). With further research on IBI, it has been revealed that elevated IBI is linked to the development and unfavorable prognoses of various diseases, including acute ischemic stroke, rheumatoid arthritis, and chronic inflammatory airway diseases (8–10). Despite the availability of various inflammatory biomarkers for identifying inflammation severity and predicting AKI progression, an ideal index to comprehensively assess the inflammatory load requires further clinical exploration and validation.

Additionally, disruption of the coagulation-fibrinolysis balance is strongly linked to the progression of AKI. COVID-19 patients with AKI exhibit significantly higher levels of D-dimer, prothrombin time (PT), and activated partial thromboplastin time (APTT) compared to their non-AKI counterparts (11). In particular, D-dimer levels strongly correlate with inflammation activity, and several studies have identified its predictive value for AKI occurrence (12–15). Furthermore, a retrospective study indicated that D-dimer may reflect the inflammatory status in patients with digestive system diseases, but no studies have addressed its relationship with inflammation in elderly AKI patients (16).

Consequently, this study aims to examine the association of IBI and D-dimer with AKI incidence in elderly Chinese patients, providing a theoretical basis for improved AKI risk stratification and early prevention.

## 2 Methods

### 2.1 Study population

**Figure 1:**
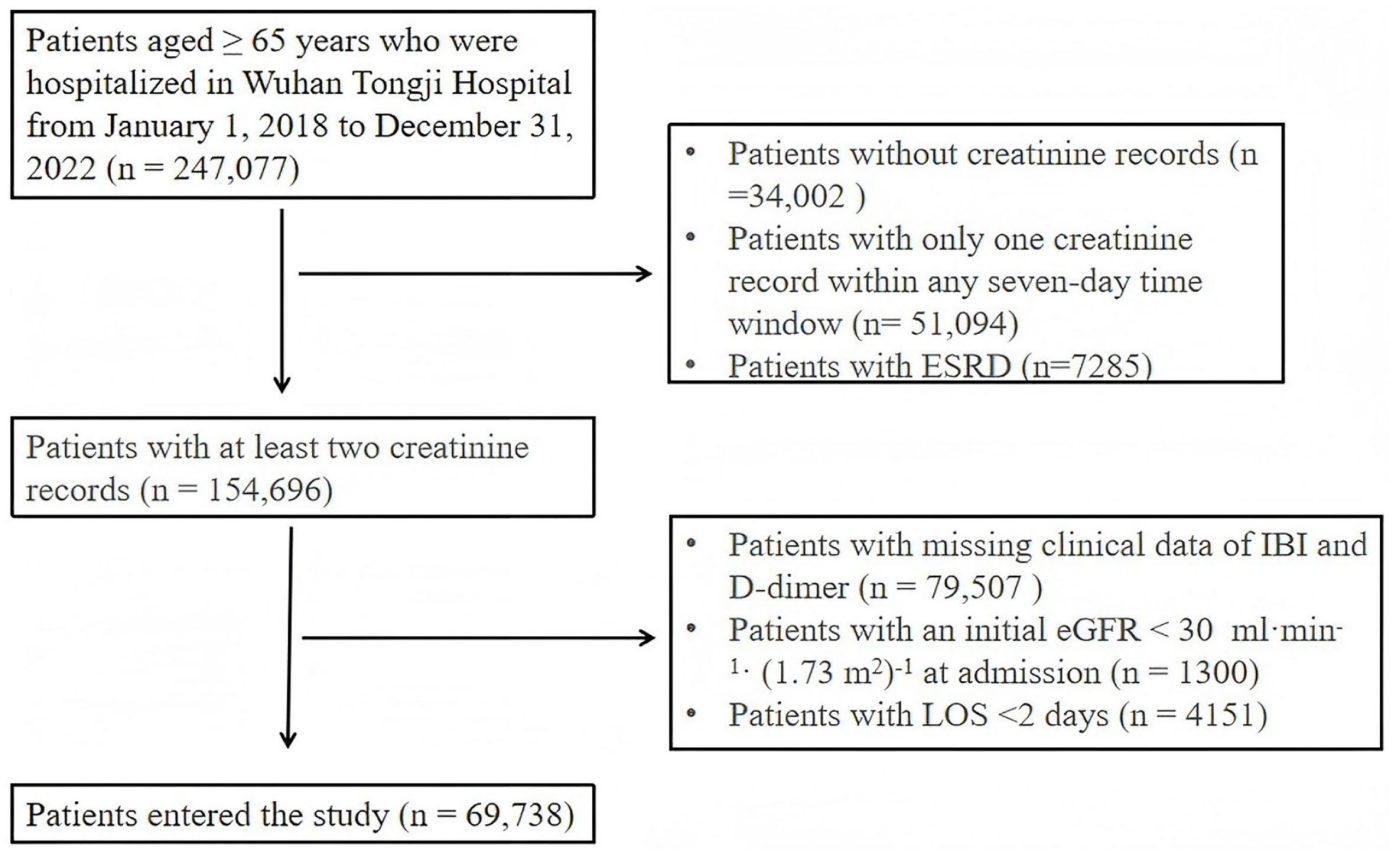
Flowchart of the study population. eGFR, Estimated glomerular filtration rate; ESRD, end-stage renal disease; IBI, inflammatory burden index. LOS, Length of hospital stay.

Elderly patients admitted to Wuhan Tongji Hospital between January 1, 2018, and December 31, 2022, were screened using the Yidu Cloud DPAP Research Center (17). Each hospitalized episode of the same patient was treated as an independent record. The flowchart (Figure 1) describes the patient selection process. An initial cohort of 247,077 elderly patients was enrolled in the study. Patients were excluded as follows: 34,002 patients without creatinine test records, 51,094 patients with only one creatinine test record within any seven-day time window, and 7,285 patients with end-stage renal disease (ESRD). In addition, 79,507 patients with missing clinical data of IBI and D-dimer, 1,300 patients with an initial estimated glomerular filtration rate (eGFR) < 30 mL·min^−1^·(1.73 m2)^−1^ at admission, and 4,151 patients with a length of hospital stay (LOS) < 2 days were excluded. Finally, a total of 69,738 patients were part of the final analysis.

### 2.2 Data source

Clinical data were taken out of the DPAP Research Center, including: 1) Demographics: Sex, age, weight, smoking status, drinking status, LOS, and in-hospital mortality; 2) Comorbidities: AKI and comorbidities (hypertension, diabetes, hyperlipidemia, chronic kidney disease (CKD), CAD, congestive heart failure (CHF), pneumonia, cerebrovascular disease, liver disease, cancer) were defined according to International Classification of Diseases, 10th Revision (ICD-10) codes; 3) Medications: Use of AKI-related drugs during hospitalization, including: Diuretics, Angiotensin-converting enzyme inhibitors/angiotensin II receptor blockers (ACEI/ARB), Nephrotoxic antibiotics, Contrast agent, Chemotherapy drugs and Nonsteroidal anti-inflammatory drugs (NSAIDs); 4) Laboratory tests: Baseline laboratory values (defined as the first measurements obtained upon admission) were collected, including high-sensitivity C-reactive protein (hsCRP), white blood cell count (WBC), neutrophil count, lymphocyte count, monocyte count, platelet count, hemoglobin, D-dimer, PT, APTT, fibrinogen, glucose, total cholesterol (TC), alanine aminotransferase (ALT), aspartate aminotransferase (AST), lactate dehydrogenase (LDH), albumin, urine protein, eGFR, uric acid, urea, bicarbonate (HCO_3_^−^), and serum potassium (K+). Compared with CRP, hsCRP is more sensitive to inflammatory responses (18). In this study, the level of hsCRP was used to assess the serum CRP level.

### 2.3 Inflammatory score

IBI = CRP (mg/L) × neutrophil count (×10^9^/L)/lymphocyte count (×10^9^/L) (7).

NLR = neutrophil count (×10^9^/L)/lymphocyte count (×10^9^/L) (19).

C-Reactive Protein to Albumin Ratio (CAR) = CRP (mg/L)/Albumin (g/L) (20).

Neutrophil-to-albumin ratio (NAR) = neutrophil count (×10^9^/L)/Albumin (g/L) (21).

Systemic immune-inflammation index (SII) = Platelet count (×10^9^/L) × neutrophil count (×10^9^/L)/lymphocyte count (×10^9^/L) (22).

Systemic inflammatory response index (SIRI) = monocyte count (×10^9^/L) × neutrophil count (×10^9^/L)/lymphocyte count (×10^9^/L) (23).

Platelet-to-lymphocyte ratio (PLR) = Platelet count (×10^9^/L)/lymphocyte count (×10^9^/L) (24).

Platelet-to-albumin ratio (PAR) = Platelet count (×10^9^/L)/Albumin (g/L) (25).

Lymphocyte-C-Reactive Protein Ratio (LCR) = lymphocyte count (×10^9^/L)/CRP (mg/L) (26).

C-reactive protein-albumin-lymphocyte (CALLY) index = Albumin (g/L) × lymphocyte count (×10^9^/L)/CRP (mg/L) (27).

### 2.4 Assessment of AKI

AKI was defined and staged according to KDIGO criteria, as follows: Stage 1, a serum creatinine increase ≥ 26.5 μmol/L within 48 h or to 1.5–1.9 times baseline within 7 days; Stage 2, an increase to 2–2.9 times baseline; Stage 3: an increase to ≥ 3 times baseline or initiation of renal replacement therapy (28). The dynamic baseline creatinine was calculated as the mean of all values from 7 days before each measurement. The earliest date meeting KDIGO criteria was defined as the AKI onset date. For multiple AKI events during hospitalization, the highest stage was recorded. Only serum creatinine was used due to missing urine output data in most cases.

### 2.5 Statistical analyses

At baseline, for continuous variables that followed a normal distribution, we reported statistics as means ± standard deviations (SD) and employed Student’s *t*-test for comparisons. In contrast, continuous non-normally distributed variables were represented as medians and interquartile ranges, with statistical comparisons made using the Mann–Whitney U test. Categorical variables were characterized by frequencies and percentages, with differences determined through chi-square tests. The multiple imputation method was used to impute the missing values.

Due to the non-normal distribution of the data, the logarithmic form of IBI (Log IBI) was used for analysis. We first performed receiver operating characteristic (ROC) analysis to evaluate the ability of IBI in predicting AKI risk among elderly patients. For biomarkers (LCR and CALLY) whose levels were inversely connected with AKI and thus yielded conventional AUC values < 0.5, we presented their predictive performance as (1 − AUC) to facilitate direct comparison with biomarkers for which higher values are associated with increased AKI risk (29, 30). Restricted cubic spline (RCS) analysis was utilized to explore the potential non-linear relationships between IBI, D-dimer, and AKI incidence.

Additionally, Logistic regression analyses were performed to assess the associations between IBI, D-dimer, and AKI risk, with 95% confidence intervals (CIs) calculated. Potential confounders were identified through univariate Logistic regression models and subsequently incorporated into multivariate Logistic regression models. Participants were partitioned into four distinct categories according to quartile distributions of Log IBI and D-dimer, with the Q1 group serving as the reference, to calculate odds ratios (ORs) and 95% CIs.

To explore the complex relationships between IBI, D-dimer, and AKI, multiplicative interaction analysis was performed, and ROC curves were constructed to assess the predictive performance of the IBI × D-dimer index. Participants were then classified into four categories based on the combined assessment of IBI and D-dimer (using medians as cutoffs), and Logistic regression was applied to analyze the association between the IBI-D-dimer combination and AKI incidence. Integrated discrimination improvement (IDI) and net reclassification improvement (NRI) were further employed to evaluate the improvement in predictive performance of combining IBI and D-dimer.

Moreover, the bootstrap method was used to validate the bidirectional mediating effects of IBI and D-dimer in AKI. Mediation analysis aimed to explore underlying mechanisms in the absence of assumptions regarding temporal precedence, rather than inferring causality.

Each covariate’s variance inflation factor (VIF) in the multicollinearity test (Additional File: Supplementary Table S2) was below 5, suggesting that there was no significant multicollinearity between the variables.

R (4.4.1) software and SPSS (25.0) software were used to perform statistical analyses. Statistical significance was set at a two-sided *p* value < 0.05.

## 3 Results

### 3.1 Baseline characteristics of participants

As shown in Table 1, among 69,738 participants, 3,755 (5.4%) cases of incident in-hospital AKI were detected. The AKI group had a median age of 72.12 (68.27, 78.20) years, included 2,473 (65.9%) males, and 1,551 (41.3%) surgical patients. Compared to the non-AKI group, the AKI group exhibited markedly elevated levels of IBI and D-dimer (170.57 (28.62, 738.14) vs. 15.29 (3.02, 119.38), *p* < 0.001; 2.18 (0.90, 5.62) vs. 0.76 (0.38, 1.73), *p* < 0.001). Additionally, the AKI group comprised a higher proportion of surgical patients (41.3% vs. 29.3%, *p* < 0.001), had a significantly longer LOS (16.00 (9.00, 27.00) vs. 10.00 (6.00, 17.00), *p* < 0.001), and showed a significantly higher in-hospital mortality rate (19.1% vs. 1.0%, *p* < 0.001).

**Table 1:** Baseline characteristics of 69,738 participants.

Characteristics	Overall (*n* = 69,738)	Non-AKI (*n* = 65,983)	AKI (*n* = 3,755)	*p*-value
Age, years	71.24 (67.75, 76.46)	71.19 (67.73, 76.36)	72.12 (68.27, 78.20)	<0.001
Gender, %				<0.001
Female	25,831 (37.0)	24,549 (37.2)	1,282 (34.1)	
Male	43,907 (63.0)	41,434 (62.8)	2,473 (65.9)	
Smoke status, %	22,062 (31.6)	21,014 (31.8)	1,048 (27.9)	<0.001
Drinking status, %	13,495 (19.4)	12,807 (19.4)	688 (18.3)	0.105
Surgery, %	20,902 (30.0)	19,351 (29.3)	1,551 (41.3)	<0.001
Underlying Disease, %				
Hypertension	28,197 (40.4)	26,628 (40.4)	1,569 (41.8)	0.086
Diabetes	13,930 (20.0)	13,100 (19.9)	830 (22.1)	0.001
Hyperlipidemia	4,601 (5.9)	4,488 (6.1)	113 (2.6)	<0.001
CKD	3,247 (4.7)	3,025 (4.6)	222 (5.9)	<0.001
CAD	14,970 (21.5)	14,055 (21.3)	915 (24.4)	<0.001
CHF	6,522 (9.4)	5,831 (8.8)	691 (18.4)	<0.001
Pneumonia	18,707 (26.8)	16,843 (25.5)	1864 (49.6)	<0.001
Liver disease	15,772 (22.6)	14,573 (22.1)	1,199 (31.9)	<0.001
Cerebrovascular disease	11,033 (15.8)	10,261 (15.6)	772 (20.6)	<0.001
Cancer	27,249 (39.1)	25,900 (39.3)	1,349 (35.9)	<0.001
WBC, ×10^9/L	6.14 (4.77, 8.17)	6.08 (4.75, 8.02)	7.83 (5.38, 11.46)	<0.001
Neutrophil, ×10^9/L	3.93 (2.81, 5.87)	3.88 (2.79, 5.70)	5.89 (3.49, 9.68)	<0.001
Lymphocyte, ×10^9/L	1.26 (0.89, 1.69)	1.28 (0.91, 1.70)	0.96 (0.60, 1.41)	<0.001
Monocyte, ×10^9/L	0.50 (0.38, 0.68)	0.50 (0.38, 0.67)	0.54 (0.37, 0.79)	<0.001
Hemoglobin, g/L	121.00 (106.00, 133.00)	121.00 (107.00, 134.00)	113.00 (94.00, 131.00)	<0.001
Platelet, ×10^9/L	194.00 (148.00, 248.00)	195.00 (150.00, 248.00)	175.00 (121.00, 235.00)	<0.001
hsCRP, mg/L	5.30 (1.40, 32.90)	5.00 (1.30, 29.65)	31.30 (6.40, 90.65)	<0.001
eGFR, ml·min-1·(1.73 m2)-1	81.30 (64.20, 90.90)	81.70 (64.90, 90.90)	72.30 (50.75, 89.35)	<0.001
Uric acid, umol/L	310.00 (244.70, 384.00)	310.00 (245.00, 382.00)	316.00 (231.50, 414.00)	0.002
Urea, mmol/L	5.90 (4.70, 7.53)	5.90 (4.64, 7.44)	7.06 (5.20, 10.10)	<0.001
Urine protein, %				<0.001
−	48,036 (68.9)	46,426 (70.4)	1,610 (42.9)	
±	8,535 (12.2)	7,986 (12.1)	549 (14.6)	
+	8,086 (11.6)	7,219 (10.9)	867 (23.1)	
++	3,981 (5.7)	3,407 (5.2)	574 (15.3)	
+++	1,088 (1.6)	935 (1.4)	153 (4.1)	
++++	12 (0.0)	10 (0.0)	2 (0.1)	
Glucose, mmol/L	5.79 (5.07, 7.31)	5.75 (5.06, 7.22)	6.76 (5.40, 9.17)	<0.001
TC, mmol/L	3.75 (3.10, 4.47)	3.77 (3.12, 4.48)	3.38 (2.68, 4.17)	<0.001
D-dimer, ug/mL	0.79 (0.39, 1.86)	0.76 (0.38, 1.73)	2.18 (0.90, 5.62)	<0.001
PT, s	13.60 (13.00, 14.40)	13.50 (13.00, 14.30)	14.40 (13.50, 15.80)	<0.001
APTT, s	37.80 (34.90, 41.50)	37.70 (34.90, 41.30)	39.70 (35.90, 45.30)	<0.001
Fibrinogen, g/L	3.58 (2.94, 4.54)	3.58 (2.95, 4.53)	3.58 (2.79, 4.70)	0.052
K+, mmol/L	4.09 (3.79, 4.36)	4.09 (3.79, 4.36)	4.11 (3.75, 4.48)	0.247
HCO3-, mmol/L	24.20 (22.40, 26.10)	24.30 (22.50, 26.10)	22.80 (20.20, 25.10)	<0.001
Albumin, g/L	38.30 (34.40, 41.50)	38.40 (34.60, 41.60)	35.00 (30.30, 39.05)	<0.001
LDH, U/L	196.00 (166.00, 242.00)	195.00 (166.00, 239.00)	233.00 (185.00, 331.00)	<0.001
ALT, U/L	16.00 (11.00, 24.00)	16.00 (11.00, 24.00)	18.00 (11.00, 33.50)	<0.001
AST, U/L	21.00 (16.00, 29.00)	21.00 (16.00, 28.00)	26.00 (18.00, 49.00)	<0.001
IBI	17.28 (3.22, 138.82)	15.29 (3.02, 119.38)	170.57 (28.62, 738.14)	<0.001
NLR	3.03 (1.94, 5.50)	2.95 (1.92, 5.22)	6.11 (2.91, 13.17)	<0.001
NAR	0.10 (0.07, 0.16)	0.10 (0.07, 0.16)	0.17 (0.10, 0.30)	<0.001
SII	587.80 (341.69, 1149.87)	575.00 (337.93, 1102.71)	1036.76 (458.46, 2281.74)	<0.001
SIRI	1.51 (0.84, 3.22)	1.47 (0.83, 3.04)	3.31 (1.31, 8.30)	<0.001
CAR	0.14 (0.03, 0.94)	0.13 (0.03, 0.84)	0.94 (0.18, 2.83)	<0.001
PLR	150.94 (106.88, 225.44)	150.00 (106.60, 222.73)	175.00 (114.17, 284.56)	<0.001
PAR	5.05 (3.85, 6.65)	5.05 (3.87, 6.63)	4.91 (3.49, 6.88)	<0.001
LCR	0.22 (0.03, 1.01)	0.25 (0.04, 1.07)	0.03 (0.01, 0.16)	<0.001
CALLY	8.50 (1.17, 40.42)	9.57 (1.33, 42.77)	1.10 (0.29, 5.75)	<0.001
Drug History, %				
Diuretic	18,497 (26.5)	15,571 (23.6)	2,926 (77.9)	<0.001
ACEI/ARB	15,367 (22.0)	14,441 (21.9)	926 (24.7)	<0.001
NSAIDs	27,153 (38.9)	25,563 (38.7)	1,590 (42.3)	<0.001
Contrast agent	27,091 (38.8)	25,481 (38.6)	1,610 (42.9)	<0.001
Nephrotoxic antibacterial drugs	10,030 (14.4)	9,173 (13.9)	857 (22.8)	<0.001
Chemotherapy drugs	8,289 (11.9)	8,015 (12.1)	274 (7.3)	<0.001
LOS, days	11.00 (6.00, 17.00)	10.00 (6.00, 17.00)	16.00 (9.00, 27.00)	<0.001
In-hospital death, %	1,371 (2.0)	655 (1.0)	716 (19.1)	<0.001

ACEI/ARB, angiotensin-converting enzyme inhibitors/angiotensin II receptor blockers; ALT, alanine aminotransferase; APTT, activated partial thromboplastin time; AST, aspartate aminotransferase; CAD, coronary artery disease; CALLY, C-reactive protein-albumin-lymphocyte; CAR, c-reactive protein to albumin ratio; CHF, congestive heart failure; CKD, chronic kidney disease; eGFR, estimated glomerular filtration rate; HCO3-, bicarbonate; hsCRP, high-sensitivity C-reactive protein; IBI, inflammatory burden index; K+, serum potassium; LCR, lymphocyte-C-reactive protein ratio; LDH, lactate dehydrogenase; LOS, length of hospital stay; NAR, neutrophil-to-albumin ratio; NLR, neutrophil-to-lymphocyte ratio; NSAIDs, nonsteroidal anti-inflammatory drugs; PAR, platelet-to-albumin ratio; PLR, platelet-to-lymphocyte ratio; PT, prothrombin time; SII, systemic immune-inflammation index; SIRI, systemic inflammatory response index; TC, total cholesterol; WBC, white blood cell.

### 3.2 Predictive efficacy of log IBI for AKI in elderly patients

**Figure 2:**
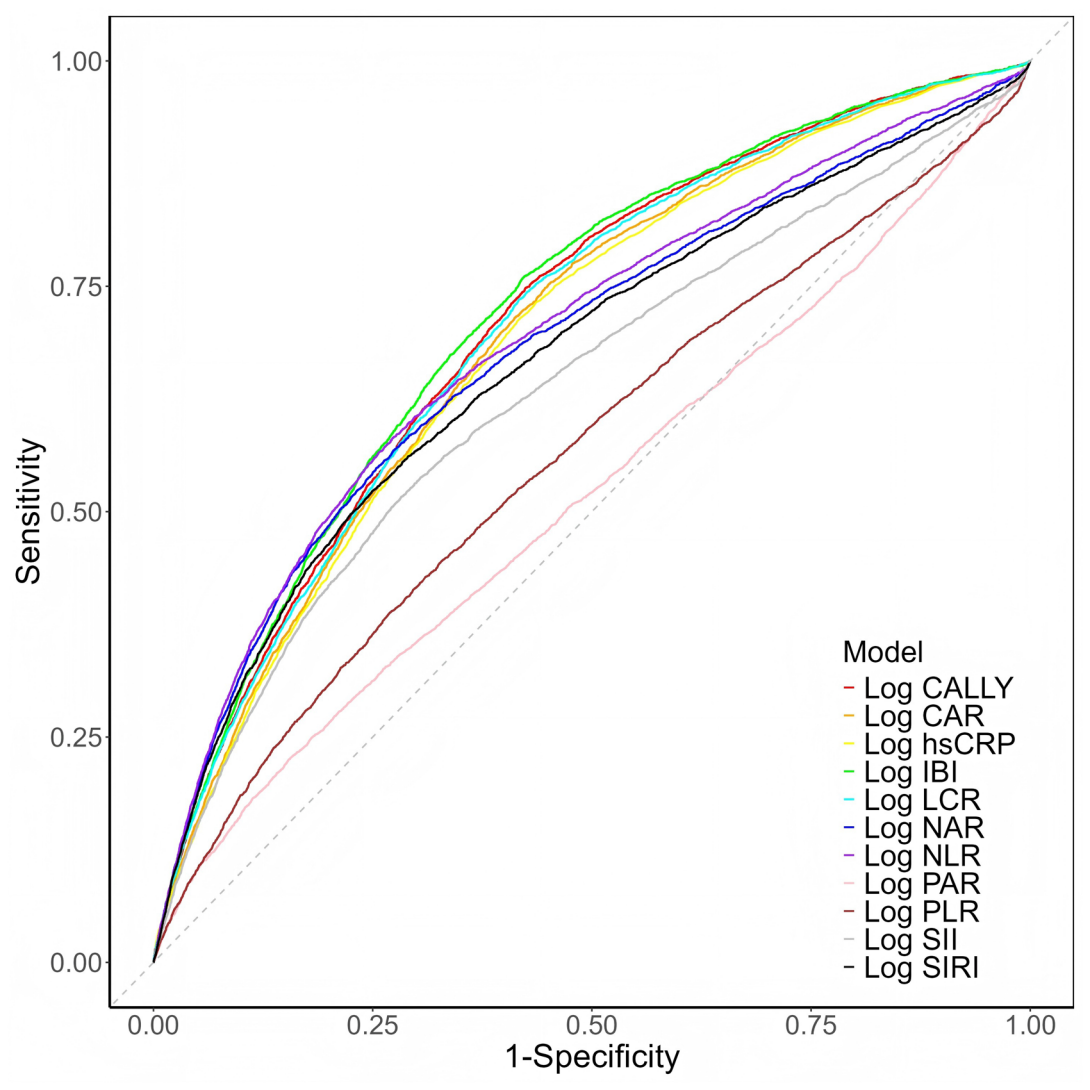
ROC curves for different inflammatory biomarkers predicting AKI in elderly patients. CALLY, C-reactive protein-albumin-lymphocyte; CAR, C-reactive protein to albumin ratio; hsCRP, high-sensitivity C-reactive protein; IBI, inflammatory burden index; LCR, lymphocyte-C-reactive protein ratio; NAR, neutrophil-to-albumin ratio; NLR, neutrophil-to-lymphocyte ratio; PAR, platelet-to-albumin ratio; PLR, platelet-to-lymphocyte ratio; SII, systemic immune-inflammation index; SIRI, systemic inflammatory response index.

Figure 2 displays ROC curves for Log IBI and other inflammatory markers. The area under the curve (AUC) for Log IBI in predicting AKI was 0.719 (95% CI 0.711–0.727, *p* < 0.001), surpassing Log hsCRP (0.692, 95% CI 0.683–0.700, *p* < 0.001), Log NLR (0.690, 95% CI 0.680–0.699, *p* < 0.001), Log CAR (0.697, 95% CI 0.689–0.706, *p* < 0.001), Log NAR (0.680, 95% CI 0.670–0.690, *p* < 0.001), Log SIRI (0.667, 95% CI 0.657–0.677, *p* < 0.001), Log SII (0.634, 95% CI 0.624–0.644, *p* < 0.001), Log PLR (0.565, 95% CI 0.555–0.575, *p* < 0.001), Log PAR (0.520, 95% CI 0.510–0.530, *p* < 0.001), Log LCR (0.706, 95% CI 0.697–0.714, *p* < 0.001), and Log CALLY (0.710, 95% CI 0.702−0.718, *p* < 0.001). DeLong’s test results showed statistically significant variations in the discriminatory performance across all markers (*p* < 0.001, Supplementary Table S3), indicating that IBI possesses superior predictive value for AKI in elderly patients compared to other inflammatory indices.

### 3.3 Associations between log IBI, D-dimer, and elderly AKI

**Figure 3:**
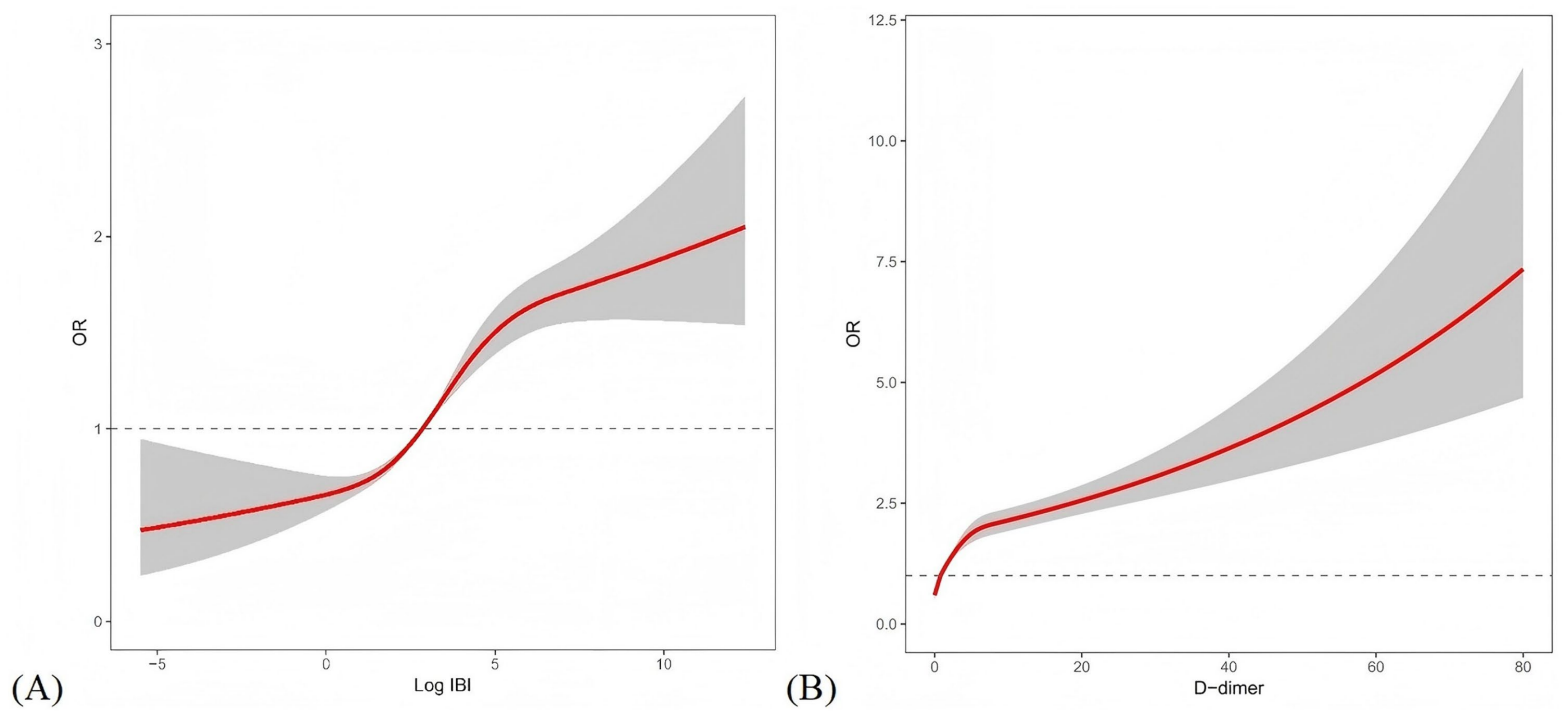
Restricted cubic spline plot: **(A)** Continuous log IBI and the risk of AKI in the entire study population; **(B)** Continuous D-dimer and the risk of AKI in the entire study population. IBI, inflammatory burden index; OR, odds ratio.

Figure 3 illustrates the dose–response relationships between Log IBI, D-dimer, and AKI risk using RCS. A significant nonlinear association was observed between continuous Log IBI and AKI risk (*p* for overall< 0.001, *p* for nonlinear<0.001, Figure 3A). Similarly, D-dimer levels exhibited a nonlinear association with increased AKI risk (*p* for overall< 0.001, *p* for nonlinear<0.001, Figure 3B).

Table 2 presents a positive correlation between Log IBI and AKI. Multivariate Logistic regression analysis, treating Log IBI as a continuous variable, showed a significant correlation with AKI risk after adjusting for confounders. When Log IBI was stratified by quartiles, participants in the top quartile had a 2.526-fold higher AKI risk (95% CI 2.193–2.918, *p* < 0.001) relative to the lowest quartile after multivariable adjustment.

**Table 2:** Logistic regression analysis on the association between the IBI and the incidence of AKI.

Variables	Model 1		Model 2		Model 3	
	OR (95% CI)	*p*-value	OR (95% CI)	*p*-value	OR (95% CI)	*p*-value
Log IBI	1.386 (1.367–1.405)	<0.001	1.230 (1.210–1.250)	<0.001	1.141 (1.121–1.161)	<0.001
Q1	Reference		Reference		Reference	
Q2	1.717 (1.479–1.998)	<0.001	1.372 (1.177–1.603)	<0.001	1.303 (1.115–1.524)	0.001
Q3	3.777 (3.305–4.331)	<0.001	2.213 (1.924–2.552)	<0.001	1.924 (1.669–2.225)	<0.001
Q4	8.168 (7.201–9.300)	<0.001	3.640 (3.180–4.181)	<0.001	2.526 (2.193–2.918)	<0.001

CI, confidence interval; IBI, inflammatory burden index; OR, odds ratio.

Model 1 was adjusted for none. Model 2 was adjusted for age, gender, smoking status, surgery, hypertension, diabetes, hyperlipidemia, CKD, CAD, CHF, pneumonia, liver disease, cerebrovascular disease, cancer, nephrotoxic antibacterial drugs, contrast agents, chemotherapy drugs, diuretics, and NSAIDs. Model 3 was adjusted for age, gender, smoking status, surgery, hypertension, diabetes, hyperlipidemia, CKD, CAD, CHF, pneumonia, liver disease, cerebrovascular disease, cancer, nephrotoxic antibacterial drugs, contrast agents, chemotherapy drugs, diuretics, NSAIDs, white blood cell count, monocyte count, platelet count, hemoglobin, LDH, uric acid, urea, urine protein, HCO_3-_, glucose, TC, PT, ALT, and AST.

Table 3 shows associations between D-dimer and AKI. Elderly patients with high D-dimer levels had a substantially increased risk of AKI (OR = 2.493, 95% CI 2.184–2.854, *p* < 0.001) relative to those with low levels. A per-unit increase in D-dimer was linked with a 3.9% elevation in AKI risk (95% CI 1.034–1.044, *p* < 0.001).

**Table 3:** Logistic regression analysis on the association between the D-dimer and the incidence of AKI.

Variables	Model 1		Model 2		Model 3	
	OR (95% CI)	*p*-value	OR (95% CI)	*p*-value	OR (95% CI)	*p*-value
D-dimer	1.078 (1.073–1.083)	<0.001	1.057 (1.052–1.062)	<0.001	1.039 (1.034–1.044)	<0.001
Q1	Reference		Reference		Reference	
Q2	1.682 (1.464–1.935)	<0.001	1.430 (1.238–1.653)	<0.001	1.331 (1.151–1.541)	<0.001
Q3	2.626 (2.309–2.993)	<0.001	1.746 (1.524–2.005)	<0.001	1.475 (1.283–1.698)	<0.001
Q4	7.223 (6.426–8.144)	<0.001	3.630 (3.198–4.131)	<0.001	2.493 (2.184–2.854)	<0.001

CI, confidence interval; OR, odds ratio.

Model 1 was adjusted for none. Model 2 was adjusted for age, gender, smoking status, surgery, hypertension, diabetes, hyperlipidemia, CKD, CAD, CHF, pneumonia, liver disease, cerebrovascular disease, cancer, nephrotoxic antibacterial drugs, contrast agents, chemotherapy drugs, diuretics, and NSAIDs. Model 3 was adjusted for age, gender, smoking status, surgery, hypertension, diabetes, hyperlipidemia, CKD, CAD, CHF, pneumonia, liver disease, cerebrovascular disease, cancer, nephrotoxic antibacterial drugs, contrast agents, chemotherapy drugs, diuretics, NSAIDs, white blood cell count, monocyte count, platelet count, hemoglobin, LDH, uric acid, urea, urine protein, HCO3-, glucose, TC, PT, ALT, and AST.

### 3.4 Predictive ability of log IBI × D-dimer for elderly AKI

**Figure 4:**
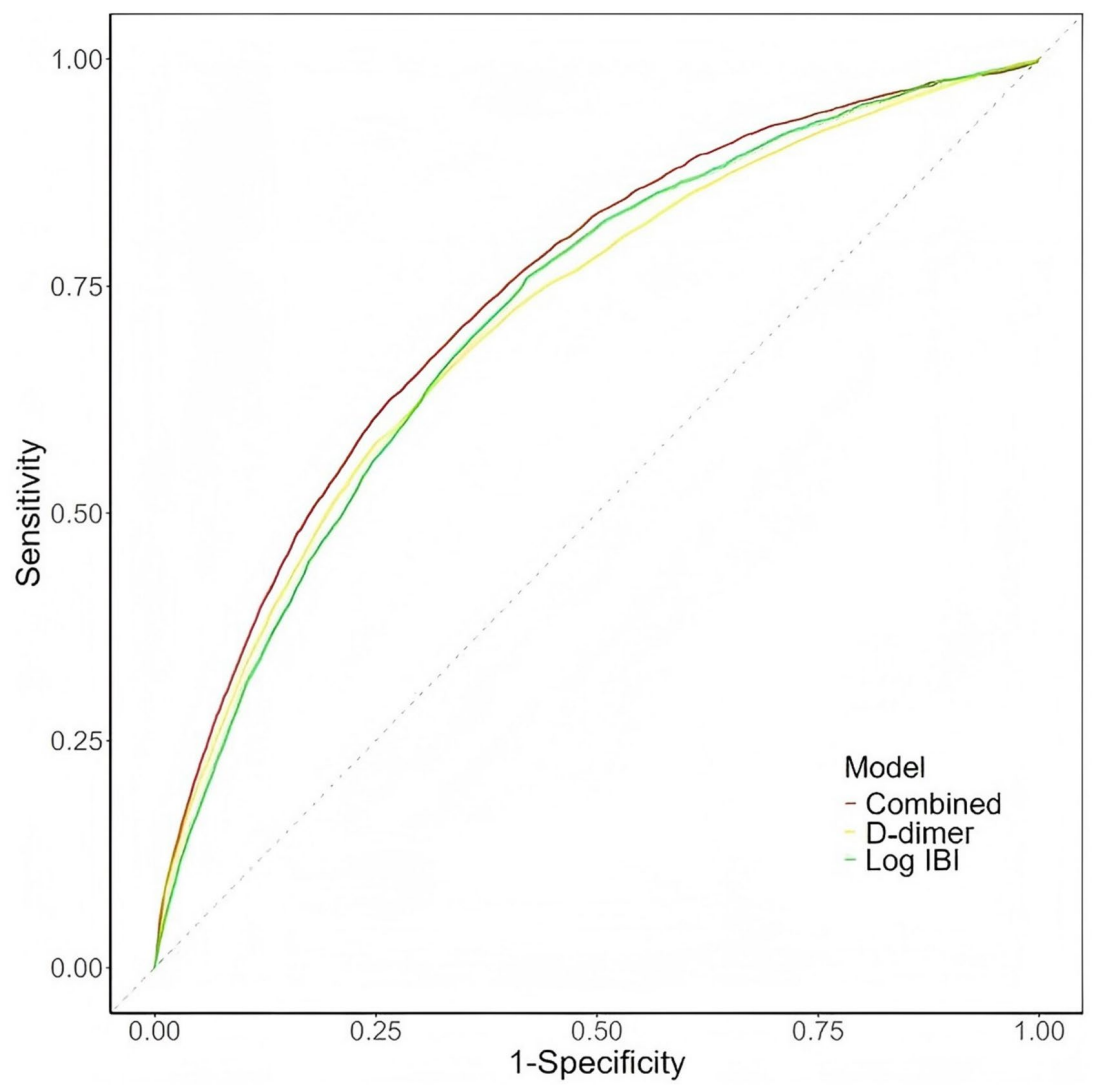
ROC curve for the combination of log IBI and D-dimer. IBI, inflammatory burden index; Combined, Log IBI × D-dimer.

Supplementary Table S4 indicates a multiplicative interaction between Log IBI and D-dimer on AKI incidence. ROC analysis (Figure 4) revealed that the AUC for Log IBI × D-dimer (0.741, 95% CI 0.733–0.749, *p* < 0.001) was superior to that for Log IBI (0.719, 95% CI 0.711–0.727, *p* < 0.001) or D-dimer (0.716, 95% CI 0.707–0.724, *p* < 0.001) alone (Supplementary Table S5).

Table 4 presents IDI and NRI to assess the incremental utility of combining Log IBI and D-dimer. Combining Log IBI with D-dimer significantly enhanced the model’s predictive performance relative to single-marker models. Specifically, compared to Log IBI alone, the combined model had an IDI of 0.005 (95% CI 0.004–0.007, *p* < 0.001) and an NRI of 0.215 (95% CI 0.184–0.246, *p* < 0.001). In comparison to D-dimer alone, it demonstrated an IDI of 0.003 (95% CI 0.002–0.004, *p* < 0.001) and an NRI of 0.303 (95% CI 0.270–0.335, *p* < 0.001).

**Table 4:** IDI and NRI to assess the incremental utility of combining Log IBI with D-dimer.

Comparison	IDI		NRI	
	OR (95% CI)	*p*-value	OR (95% CI)	*p*-value
Log IBI × D-dimer vs. Log IBI	0.005(0.004–0.007)	<0.001	0.215 (0.184–0.246)	<0.001
Log IBI × D-dimer vs. D-dimer	0.003 (0.002–0.004)	<0.001	0.303 (0.270–0.335)	<0.001

Adjusted for age, gender, smoking status, surgery, hypertension, diabetes, hyperlipidemia, CKD, CAD, CHF, pneumonia, liver disease, cerebrovascular disease, cancer, nephrotoxic antibacterial drugs, contrast agents, chemotherapy drugs, diuretics, NSAIDs, white blood cell count, monocyte count, platelet count, hemoglobin, LDH, uric acid, urea, urine protein, HCO3-, glucose, TC, PT, ALT, and AST. CI, confidence interval; IBI, inflammatory burden index; IDI, integrated discrimination improvement; NRI, net reclassification improvement; OR, odds ratio.

### 3.5 Association of combined IBI-D-dimer index with AKI incidence

Table 5 presents associations between Log IBI-D-dimer combinations and AKI incidence across three Logistic regression models. In the final multivariate model (Model 3), participants with both high Log IBI (≥ median) and high D-dimer (≥ median) had the highest AKI risk (OR = 2.640, 95% CI 2.328–3.002, *p* < 0.001) relative to those with low levels of both biomarkers.

**Table 5:** Association between combined Log IBI and D-dimer and the incidence of AKI.

Groups	Model 1		Model 2		Model 3	
	OR (95% CI)	*p*-value	OR (95% CI)	*p*-value	OR (95% CI)	*p*-value
Log IBI < median and D-dimer < median	Reference		Reference		Reference	
Log IBI < median and D-dimer ≥ median	2.648 (2.289–3.065)	<0.001	1.988 (1.709–2.314)	<0.001	1.718 (1.473–2.005)	<0.001
Log IBI ≥ median and D-dimer < median	3.440 (2.997–3.952)	<0.001	2.219 (1.923–2.565)	<0.001	1.963 (1.697–2.272)	<0.001
Log IBI ≥ median and D-dimer≥ median	7.827 (7.002–8.776)	<0.001	3.759 (3.333–4.250)	<0.001	2.640 (2.328–3.002)	<0.001

CI, confidence interval; IBI, inflammatory burden index; OR, odds ratio.

Model 1 was adjusted for none. Model 2 was adjusted for age, gender, smoking status, surgery, hypertension, diabetes, hyperlipidemia, CKD, CAD, CHF, pneumonia, liver disease, cerebrovascular disease, cancer, nephrotoxic antibacterial drugs, contrast agents, chemotherapy drugs, diuretics, and NSAIDs. Model 3 was adjusted for age, gender, smoking status, surgery, hypertension, diabetes, hyperlipidemia, CKD, CAD, CHF, pneumonia, liver disease, cerebrovascular disease, cancer, nephrotoxic antibacterial drugs, contrast agents, chemotherapy drugs, diuretics, NSAIDs, white blood cell count, monocyte count, platelet count, hemoglobin, LDH, uric acid, urea, urine protein, HCO3-, glucose, TC, PT, ALT, and AST.

### 3.6 Subgroup analysis and interaction analysis

**Figure 5:**
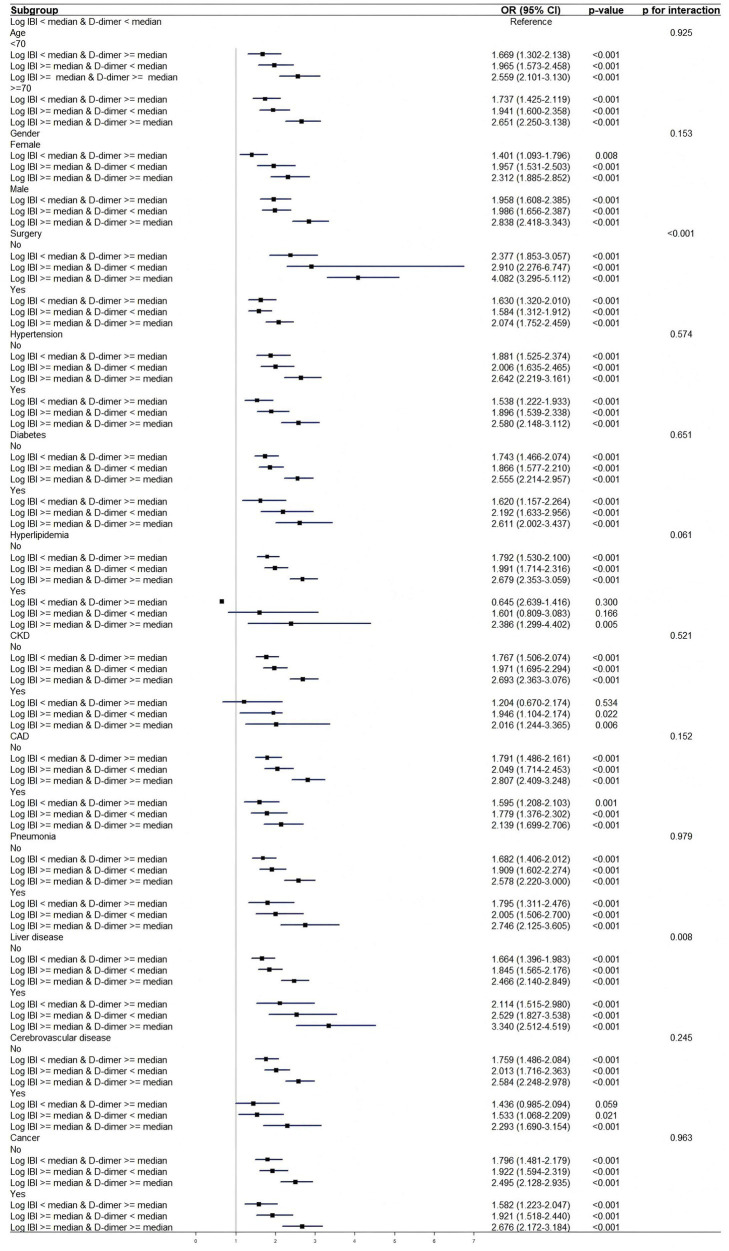
Subgroup analysis and interactions. CAD, Coronary Artery Disease; CKD, Chronic Kidney Disease; IBI, Inflammatory Burden Index; OR, odds ratio.

Figure 5 shows subgroup and interaction analyses stratified by age, sex, surgery, hypertension, diabetes, hyperlipidemia, CKD, CAD, pneumonia, liver disease, cerebrovascular disease, and cancer. Significant interactions were identified exclusively in the surgical (*p* < 0.001) and liver disease subgroups (*p* = 0.008), whereas no interactions were detected in the remaining subgroups (*p* > 0.05). Corresponding numerical results are provided in Supplementary Table S6.

### 3.7 Mediation analysis

**Figure 6:**
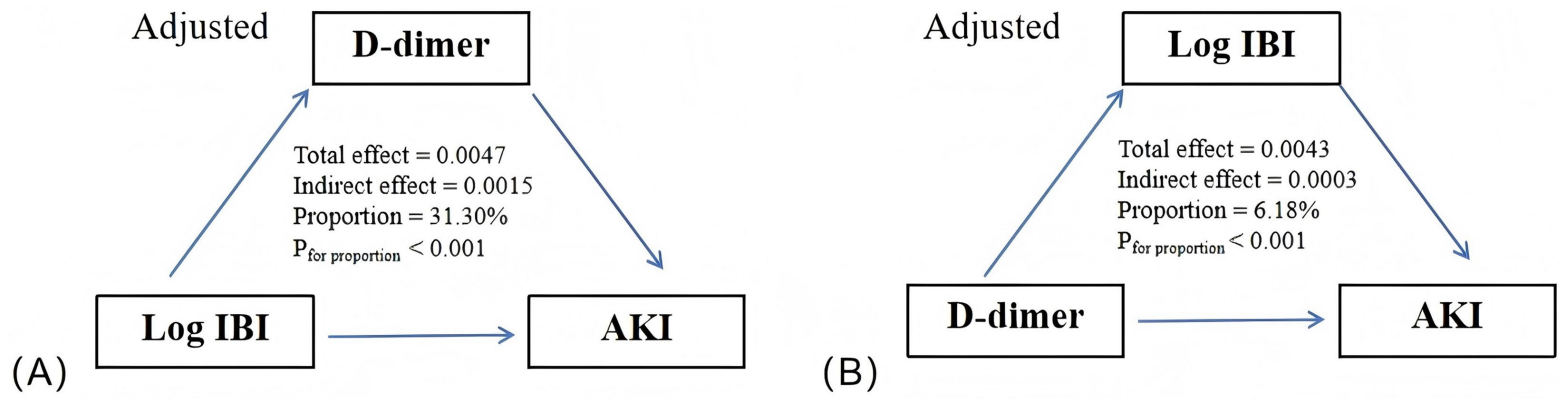
Mediation effects: **(A)** Mediation effect of D-dimer on the association between Log IBI and the risk of AKI; **(B)** Mediation effect of Log IBI on the association between D-dimer and the risk of AKI. Adjusted for age, gender, smoking status, surgery, hypertension, diabetes, hyperlipidemia, CKD, CAD, CHF, pneumonia, liver disease, cerebrovascular disease, cancer, nephrotoxic antibacterial drugs, contrast agents, chemotherapy drugs, diuretics, NSAIDs, white blood cell count, monocyte count, platelet count, hemoglobin, LDH, uric acid, urea, urine protein, HCO3-, glucose, TC, PT, ALT, and AST. AKI, acute kidney injury; IBI, inflammatory burden index.

Figure 6 illustrates the potential mediating effects of elevated Log IBI and D-dimer on AKI incidence. Bootstrap analysis controlling for confounders revealed that D-dimer mediated 31.30% (*p* < 0.001) of the Log IBI-AKI association, while Log IBI mediated 6.18% (*p* < 0.001) of the D-dimer-AKI association. This bidirectional mediation reflects intertwined relationships between inflammation and coagulation disorders, rather than strict causal pathways.

## 4 Discussion

In a cohort of 69,738 elderly patients from Wuhan Tongji Hospital, our analysis confirmed that elevated IBI and D-dimer were independently linked to increased AKI risk, with a bidirectional partial mediation between these two markers. Notably, the combined use of IBI and D-dimer increased the predictive accuracy for AKI in elderly patients. According to the literature search, this study makes a pioneering contribution by investigating the independent predictive value of IBI for AKI in the elderly and elucidating the complex relationship among IBI, D-dimer, and AKI.

Inflammation is a well-established driver in the pathogenesis of AKI (31). Chronic inflammation serves as a common risk factor for multiple diseases, including kidney disease, cardiovascular disease, diabetes, and cancer (32). Elderly people typically exhibit higher baseline inflammation levels due to factors such as cellular senescence, mitochondrial dysfunction, NLRP3 inflammasome activation, and chronic infections (33). When the body encounters pathogenic factors such as infection, trauma, or shock, damaged cells and immune cells release pro-inflammatory cytokines [e.g., tumor necrosis factor (TNF), interleukin-6 (IL-6)], recruiting mononuclear macrophages and neutrophils to the kidney and triggering inflammatory cascades that lead to renal tubular injury (34). Meanwhile, the excessive inflammatory state further damages endothelial cells (ECs), which leads to persistent renal vasoconstriction through mechanisms such as increased secretion of vasoconstrictors and reduced release of vasodilators, exacerbating renal injury (35). Numerous previous studies have confirmed that inflammatory markers such as NLR, SII, and SIRI possess early predictive value for AKI (6, 36, 37). As a novel inflammatory biomarker, IBI not only accounts for systemic inflammation (CRP) but also incorporates immune response balance (NLR), thereby enabling a more comprehensive assessment of immune-mediated inflammation in AKI. In addition, compared with emerging predictive markers like C-C chemokine ligand 14 (CCL14), kidney injury molecule-1 (KIM-1), soluble urokinase plasminogen activator receptor (suPAR), and neutrophil gelatinase-associated lipocalin (NGAL), IBI has the advantages of low cost, high prevalence, and short testing time (38). In this study, by comparing various more readily available inflammatory markers, we found that IBI had superior predictive capacity for AKI in the elderly and emerged as an independent predictor for AKI. Using IBI as an inflammatory marker, further analysis confirmed a positive relationship between IBI levels and AKI risk, which aligns with the results of existing literature (39). Although the observed AUC for IBI was modest at 0.719, it may serve as a useful adjunctive tool for risk stratification, facilitating the early identification of elderly patients at increased risk of AKI and enabling closer clinical monitoring and earlier preventive intervention.

Inflammation can disrupt the coagulation balance. During excessive inflammatory states, upregulated inflammatory cytokines (e.g., CRP, IL-6) stimulate neutrophils and macrophages via IL-1β to release tissue factor (TF), triggering platelet hyperreactivity and promoting fibrinogen biosynthesis (40, 41). Additionally, the vascular endothelium is a central driver of the inflammation-coagulation axis. Following the activation of NLRP3 inflammasome, ECs exhibit a pro-thrombotic phenotype, characterized by upregulated adhesion molecules (e.g., P-selectin, ICAM-1, vascular cell adhesion molecule 1), increased vascular permeability, and dysregulated secretion of pro-coagulant factors alongside reduced endogenous anticoagulants, leading to aberrant activation of coagulation and fibrinolysis (42). This suggests that coagulation abnormalities may partially mediate the relationship between inflammation and AKI. Our study showed that D-dimer, a representative marker of coagulation abnormalities, exhibited a significant mediating effect in the relationship between IBI and AKI.

D-dimer, a soluble fibrin degradation product, is a biomarker of the coordinated activation of coagulation and fibrinolysis systems (43). Elevated D-dimer typically indicates an imbalance between coagulation and fibrinolysis and is linked to poor prognosis of critically ill patients (44). A retrospective study indicated that increased D-dimer levels were linked to the incidence and poor outcomes of contrast-induced AKI (CI-AKI) (13). Similarly, in a study of patients undergoing living donor liver transplantation, a marked difference in AKI incidence was observed between the high and normal D-dimer groups (15). These findings are in line with the results of our study.

At present, the underlying mechanisms of elevated D-dimer levels in AKI development remain unclear, but D-dimer elevation may induce increased inflammatory markers through several interrelated molecular mechanisms. As an acute-phase reactant, abnormally elevated D-dimer can activate neutrophils and monocytes through Toll-like receptor pathways and induce the secretion of pro-inflammatory cytokines such as IL-6 and IL-8, thereby triggering inflammatory cascades (45). A retrospective study in patients with COVID-19 demonstrated that elevated D-dimer can lead to EC dysfunction and complement system activation, exacerbating the inflammatory response (46). This suggests that IBI, a representative indicator of inflammatory response, partially mediates the link between D-dimer and AKI. Our study showed that Log IBI mediated 6.18% of the association between D-dimer and AKI.

The bidirectional association between IBI and D-dimer reflects the dysregulation of the inflammation-coagulation axis in AKI. Excessive inflammatory response activates the coagulation system via TF, leading to renal microcirculatory dysfunction and exacerbating renal ischemia and hypoxia, whereas coagulation abnormalities activate protease-activated receptors (PARs) and promote inflammatory responses mediated by the NF-κB pathway, creating a vicious cycle (47). Notably, mediation analysis revealed that the mediation proportion from Log IBI to D-dimer (31.30%) was substantially larger than that in the reverse direction (6.18%). This asymmetry suggests that, although inflammation and coagulation abnormalities are closely intertwined in the pathogenesis of AKI, the predominant direction of influence is from inflammation to coagulation dysfunction, which aligns with the underlying pathophysiology of AKI. As an early response to injury, inflammation may trigger abnormal activation of the coagulation cascade, thereby exacerbating kidney injury. Moreover, this asymmetry also helps mitigate concerns regarding reverse causality, providing robust support for the reliability of the observed bidirectional association.

In our analysis of 69,738 elderly patients, statistical improvements in IDI and NRI for the combined IBI × D-dimer index demonstrated incremental predictive value for integrating IBI and D-dimer into AKI risk stratification. Further analysis revealed that the group with high IBI and high D-dimer had a 2.640-fold higher AKI risk than the group with low IBI and low D-dimer. These findings help to confirm the predictive value of combined IBI and D-dimer analyses for AKI, allowing for more accurate identification of high-risk individuals at admission. Our study preliminarily reveals the intricate interplay between inflammation and coagulation in elderly AKI.

Further subgroup and interaction analyses revealed significant between-group heterogeneity in the effects of Log IBI and D-dimer on AKI: in the subgroups of patients with a history of surgery during hospitalization (*p* < 0.001) and liver disease (*p* = 0.008), the interaction between the two was statistically significant. These findings support the implementation of targeted monitoring of IBI and D-dimer in these two subgroups to optimize AKI risk stratification. This result may be attributed to the following factors. Firstly, AKI patients had a significantly higher rate of surgical exposure compared with the non-AKI population (41.3% vs. 29.3%, *p* < 0.001). Studies have found that D-dimer increases in postoperative patients, suggesting that surgical stress may synergistically amplify the effects of the inflammation-coagulation axis (13, 48). Secondly, liver disease, particularly cirrhosis, represents a major risk factor for AKI (49). In liver disease, activation of the NLRP3 inflammasome drives the secretion of pro-inflammatory cytokines, leading to an inflammatory response (50). As the disease progresses, excessive inflammation can activate the immune system, potentially leading to organ failure (50–52). D-dimer is elevated in chronic liver disease, with its levels closely linked to disease severity and prognosis (52). A study has shown that D-dimer levels can predict the development of AKI after liver transplantation (53). Our research found that abnormal activation of the inflammatory and coagulation systems mutually reinforces each other in liver disease, creating a vicious cycle consistent with the result of the previous study (54).

Several limitations are also present in this study. Firstly, unavoidable biases may exist in the data due to the single-center retrospective nature of this study. Secondly, the study did not collect data on anticoagulant use (e.g., warfarin, low-molecular-weight heparin), which may have led to a biased assessment of D-dimer’s predictive value. Finally, given the observational design of the study, causal relationships between IBI, D-dimer, and AKI cannot be established and need to be further explored.

## 5 Conclusion

Both IBI and D-dimer were identified as independent risk factors for AKI in older individuals. Their combined elevation demonstrated a synergistic effect, significantly increasing AKI risk. Consequently, these results suggest the combined use of both biomarkers for improved AKI risk stratification, particularly in elderly patients with liver disease or those undergoing surgery.

## Data Availability

The raw data supporting the conclusions of this article will be made available by the authors, without undue reservation.
