# Supplementary material for: Association between inflammatory burden index combined with D-dimer and acute kidney injury in elderly patients
Source: Front Med (Lausanne). 2026 Apr 28;13:1764718. doi: 10.3389/fmed.2026.1764718 (PMC13160860; doi:10.3389/fmed.2026.1764718)
Supplement: Supplementary file 1 [file Data_Sheet_1.docx]

**Supplemental Material**

Table S1. Baseline characteristics according to the Log IBI quartiles.

| Characteristics | Log IBI Q1 ~ 1.17 | Log IBI Q2 1.17 ~ 2.85 | Log IBI Q3 2.85 ~ 4.93 | Log IBI Q4 4.93 ~ | P-value |
| --- | --- | --- | --- | --- | --- |
| Age, years | 70.22 (67.33, 74.71) | 71.11 (67.68, 76.15) | 71.63 (67.93, 77.19) | 72.15 (68.23, 77.99) | <0.001 |
| Gender, % |  |  |  |  | <0.001 |
| Female | 7132 (40.9) | 6852 (39.3) | 6292 (36.1) | 5555 (31.9) |  |
| Male | 10301 (59.1) | 10583 (60.7) | 11143 (63.9) | 11880 (68.1) |  |
| Smoke status, % | 4855 (27.8) | 5595 (32.1) | 5796 (33.2) | 5816 (33.4) | <0.001 |
| Drinking status, % | 3109 (17.8) | 3518 (20.2) | 3498 (20.1) | 3370 (19.3) | <0.001 |
| Surgery, % | 6206 (35.6) | 5048 (29.0) | 4986 (28.6) | 4662 (26.7) | <0.001 |
| Underlying Disease, % |  |  |  |  |  |
| Hypertension | 7291 (41.8) | 7631 (43.8) | 6859 (39.3) | 6416 (36.8) | <0.001 |
| Diabetes | 3328 (19.1) | 3730 (21.4) | 3479 (20.0) | 3393 (19.5) | <0.001 |
| Hyperlipidemia | 1496 (8.6) | 1456 (8.4) | 826 (4.7) | 473 (2.7) | <0.001 |
| CKD | 683 (3.9) | 945 (5.4) | 913 (5.2) | 706 (4.0) | <0.001 |
| CAD | 4224 (24.2) | 4113 (23.6) | 3570 (20.5) | 3063 (17.6) | <0.001 |
| CHF | 1256 (7.2) | 1707 (9.8) | 1806 (10.4) | 1753 (10.1) | <0.001 |
| Pneumonia | 1827 (10.5) | 3237 (18.6) | 5360 (30.7) | 8283 (47.5) | <0.001 |
| Liver disease | 3170 (18.2) | 3609 (20.7) | 4295 (24.6) | 4698 (26.9) | <0.001 |
| Cerebrovascular disease | 2583 (14.8) | 2739 (15.7) | 2799 (16.1) | 2912 (16.7) | <0.001 |
| Cancer | 6562 (37.6) | 7013 (40.2) | 7133 (40.9) | 6541 (37.5) | <0.001 |
| WBC, ×10^9/L | 5.14 (4.18, 6.19) | 5.78 (4.64, 7.12) | 6.41 (4.94, 8.32) | 8.61 (6.33, 11.76) | <0.001 |
| Neutrophil, ×10^9/L | 2.88 (2.21, 3.65) | 3.58 (2.72, 4.65) | 4.33 (3.12, 5.98) | 6.77 (4.65, 9.85) | <0.001 |
| Lymphocyte, ×10^9/L | 1.54 (1.21, 1.94) | 1.39 (1.05, 1.80) | 1.19 (0.86, 1.58) | 0.88 (0.59, 1.25) | <0.001 |
| Monocyte, ×10^9/L | 0.43 (0.34, 0.54) | 0.49 (0.38, 0.62) | 0.54 (0.40, 0.71) | 0.63 (0.43, 0.88) | <0.001 |
| Hemoglobin, g/L | 126.00 (115.00, 137.00) | 124.00 (111.00, 135.00) | 118.00 (103.00, 131.00) | 113.00 (96.00, 127.00) | <0.001 |
| Platelet, ×10^9/L | 184.00 (147.00, 224.00) | 195.00 (151.00, 243.00) | 199.00 (149.00, 259.00) | 202.00 (145.00, 275.00) | <0.001 |
| hsCRP, mg/L | 0.70 (0.40, 1.10) | 2.80 (1.80, 4.40) | 12.40 (7.10, 23.30) | 74.90 (44.40, 125.00) | <0.001 |
| eGFR, ml·min-1· (1.73 m2)-1 | 82.50 (68.30, 90.70) | 80.20 (63.90, 90.20) | 81.50 (63.20, 91.20) | 80.80 (60.50, 91.50) | <0.001 |
| Uric acid, umol/L | 317.00 (261.00, 380.00) | 327.00 (265.00, 397.00) | 310.00 (241.00, 388.20) | 280.00 (208.00, 364.00) | 0.002 |
| Urea, mmol/L | 5.82 (4.79, 7.13) | 5.90 (4.70, 7.31) | 5.80 (4.50, 7.50) | 6.20 (4.70, 8.52) | <0.001 |
| Urine protein, % |  |  |  |  | <0.001 |
| - | 48036 (68.9) | 14765 (84.7) | 13249 (76.0) | 11673 (67.0) |  |
| ± | 8535 (12.2) | 1218 (7.0) | 1779 (10.2) | 2305 (13.2) |  |
| + | 8086 (11.6) | 853 (4.9) | 1363 (7.8) | 2111 (12.1) |  |
| ++ | 3981 (5.7) | 425 (2.4) | 753 (4.3) | 1042 (6.0) |  |
| +++ | 1088 (1.6) | 170 (1.0) | 287 (1.6) | 299 (1.7) |  |
| ++++ | 12 (0.0) | 2 (0.0) | 4 (0.0) | 5 (0.0) |  |
| Glucose, mmol/L | 5.40 (4.91, 6.45) | 5.67 (5.03, 7.06) | 5.86 (5.12, 7.44) | 6.43 (5.39, 8.28) | <0.001 |
| TC, mmol/L | 3.90 (3.25, 4.60) | 3.92 (3.26, 4.64) | 3.74 (3.09, 4.44) | 3.44 (2.82, 4.14) | <0.001 |
| D-dimer, ug/mL | 0.39 (0.27, 0.71) | 0.59 (0.35, 1.17) | 1.04 (0.52, 2.11) | 1.94 (0.97, 3.93) | <0.001 |
| PT, s | 13.30 (12.80, 13.80) | 13.30 (12.80, 14.00) | 13.60 (13.10, 14.40) | 14.30 (13.50, 15.20) | <0.001 |
| APTT, s | 36.50 (34.10, 39.00) | 36.80 (34.30, 39.70) | 38.20 (35.20, 41.80) | 41.00 (37.00, 45.90) | <0.001 |
| Fibrinogen, g/L | 2.97 (2.62, 3.36) | 3.45 (2.98, 3.97) | 3.97 (3.22, 4.76) | 4.89 (3.74, 6.07) | <0.001 |
| K+, mmol/L | 4.11 (3.87, 4.34) | 4.11 (3.83, 4.36) | 4.08 (3.76, 4.38) | 4.04 (3.69, 4.38) | <0.001 |
| HCO3-, mmol/L | 24.60 (23.00, 26.20) | 24.40 (22.70, 26.10) | 24.20 (22.20, 26.10) | 23.50 (21.40, 25.70) | <0.001 |
| Albumin, g/L | 40.80 (38.40, 43.30) | 39.70 (36.80, 42.40) | 37.30 (33.80, 40.30) | 33.70 (30.00, 37.40) | <0.001 |
| LDH, U/L | 181.00 (158.00, 209.00) | 191.00 (165.00, 227.00) | 204.00 (170.00, 255.00) | 223.00 (178.00, 304.00) | <0.001 |
| ALT, U/L | 15.00 (11.00, 21.00) | 15.00 (11.00, 23.00) | 15.00 (10.00, 25.00) | 17.00 (11.00, 31.00) | <0.001 |
| AST, U/L | 20.00 (16.00, 25.00) | 20.00 (16.00, 26.00) | 21.00 (16.00, 31.00) | 23.00 (17.00, 39.00) | <0.001 |
| Drug History, % |  |  |  |  |  |
| Diuretic | 2357 (13.5) | 3649 (20.9) | 5284 (30.3) | 7207 (41.3) | <0.001 |
| ACEI/ARB | 4359 (25.0) | 4500 (25.8) | 3690 (21.2) | 2818 (16.2) | <0.001 |
| NSAIDs | 7301 (41.9) | 6715 (38.5) | 6594 (37.8) | 6543 (37.5) | <0.001 |
| Contrast agent | 7003 (40.2) | 6869 (39.4) | 6948 (39.9) | 6271 (36.0) | <0.001 |
| Nephrotoxic antibacterial drugs | 2093 (12.0) | 2073 (11.9) | 2637 (15.1) | 3227 (18.5) | <0.001 |
| Chemotherapy drugs | 2102 (12.1) | 2273 (13.0) | 2351 (13.5) | 1563 (9.0) | <0.001 |
| AKI max stage, % |  |  |  |  | <0.001 |
| 1 | 203 (1.2) | 335 (1.9) | 697 (4.0) | 1236 (7.1) |  |
| 2 | 45 (0.3) | 71 (0.4) | 179 (1.0) | 473 (2.7) |  |
| 3 | 27 (0.2) | 61 (0.3) | 119 (0.7) | 309 (1.8) |  |
| LOS, days | 9.00 (5.00, 14.00) | 9.00 (6.00, 15.00) | 12.00 (7.00, 18.00) | 13.00 (8.00, 20.00) | <0.001 |
| In-hospital death, % | 31 (0.2) | 79 (0.5) | 280 (1.6) | 981 (5.6) | <0.001 |

**Abbreviations**: ACEI/ARB, angiotensin-converting enzyme inhibitors/angiotensin II receptor blockers; ALT, alanine aminotransferase; APTT, activated partial thromboplastin time; AST, aspartate aminotransferase; CAD, coronary artery disease; CHF, congestive heart failure; CKD, chronic kidney disease; eGFR, estimated glomerular filtration rate; HCO3-, bicarbonate; hsCRP, high-sensitivity C-reactive protein; IBI, inflammatory burden index; K+, serum potassium; LDH, lactate dehydrogenase; LOS, length of hospital stay; NSAIDs, nonsteroidal anti-inflammatory drugs; PT, prothrombin time; TC, total cholesterol; WBC, white blood cell.

Table S2. Collinearity Statistics.

| Variables | VIF |
| --- | --- |
| Age | 1.198 |
| Gender | 1.329 |
| Smoke status | 1.252 |
| Surgery | 1.617 |
| Hypertension | 1.234 |
| Diabetes | 1.343 |
| Hyperlipidemia | 1.051 |
| CKD | 1.097 |
| CAD | 1.270 |
| CHF | 1.201 |
| Pneumonia | 1.313 |
| Liver disease | 1.113 |
| Cerebrovascular disease | 1.117 |
| Cancer | 1.299 |
| Log IBI | 1.443 |
| D-dimer | 1.501 |
| WBC | 1.742 |
| Monocyte | 1.691 |
| Hemoglobin | 1.287 |
| Platelet | 1.149 |
| Uric acid | 1.374 |
| Urea | 1.468 |
| Urine protein | 1.565 |
| Glucose | 1.321 |
| TC | 1.330 |
| PT | 1.126 |
| HCO3- | 1.156 |
| LDH | 1.531 |
| ALT | 3.819 |
| AST | 4.323 |
| Diuretic | 1.145 |
| NSAIDs | 1.253 |
| Contrast agent | 1.256 |
| Nephrotoxic antibacterial drugs | 1.115 |
| Chemotherapy drugs | 1.213 |

**Abbreviations**: ALT, alanine aminotransferase; AST, aspartate aminotransferase; CAD, coronary artery disease; CHF, congestive heart failure; CKD, chronic kidney disease; HCO3-, bicarbonate; IBI, inflammatory burden index; LDH, lactate dehydrogenase; NSAIDs, nonsteroidal anti-inflammatory drugs; PT, prothrombin time; TC, total cholesterol; WBC, white blood cell.

Table S3. Comparison of AUC values between Log IBI and other inflammatory markers.

| Variables | AUC | 95% CI low | 95% CI upp | Best threshold | Sensitivity | Specificity | P for the difference in AUC |
| --- | --- | --- | --- | --- | --- | --- | --- |
| Log IBI | 0.719 | 0.711 | 0.727 | 3.309 | 0.759 | 0.579 |  |
| Log hsCRP | 0.692 | 0.683 | 0.700 | 2.218 | 0.712 | 0.585 | <0.001 |
| Log NLR | 0.690 | 0.680 | 0.699 | 1.584 | 0.581 | 0.728 | <0.001 |
| Log CAR | 0.697 | 0.689 | 0.706 | -1.474 | 0.715 | 0.588 | <0.001 |
| Log NAR | 0.680 | 0.670 | 0.690 | -1.908 | 0.568 | 0.726 | <0.001 |
| Log SIRI | 0.667 | 0.657 | 0.677 | 1.113 | 0.524 | 0.750 | <0.001 |
| Log SII | 0.634 | 0.624 | 0.644 | 6.899 | 0.516 | 0.718 | <0.001 |
| Log PLR | 0.565 | 0.555 | 0.575 | 5.368 | 0.388 | 0.730 | <0.001 |
| Log PAR | 0.520 | 0.510 | 0.530 | 1.197 | 0.225 | 0.846 | <0.001 |
| Log LCR | 0.706 | 0.697 | 0.714 | -1.948 | 0.736 | 0.583 | <0.001 |
| Log CALLY | 0.710 | 0.702 | 0.718 | 1.722 | 0.749 | 0.575 | <0.001 |

**Abbreviations**: AUC, area under the curve; CAR, C-reactive protein to albumin ratio; CI, confidence interval; hsCRP, high-sensitivity C-reactive protein; IBI, inflammatory burden index; NAR, neutrophil-to-albumin ratio; NLR, neutrophil-to-lymphocyte ratio; PLR, platelet-to-lymphocyte ratio; SII, systemic immune-inflammation index; SIRI, systemic inflammatory response index.

Table S4. The multiplicative interaction of Log IBI and D-dimer on AKI risk in elderly patients.

| Multiplicative interaction |  |
| --- | --- |
| *P*-value | <0.001 |

Table S5. Comparison of AUC values between the combined index of Log IBI and D-dimer and the individual indices ( Log IBI and D-dimer ).

| Variables | AUC | 95% CI low | 95% CI upp | Best threshold | Sensitivity | Specificity | P for the difference in AUC |
| --- | --- | --- | --- | --- | --- | --- | --- |
| Log IBI × D-dimer | 0.741 | 0.733 | 0.749 | 6.225 | 0.627 | 0.733 |  |
| Log IBI | 0.719 | 0.711 | 0.727 | 3.309 | 0.759 | 0.579 | <0.001 |
| D-dimer | 0.716 | 0.707 | 0.724 | 1.725 | 0.579 | 0.749 | <0.001 |

**Abbreviations**: AUC, area under the curve; CI, confidence interval; IBI, inflammatory burden index

Table S6. Subgroup analyses of the association of the Log IBI and D-dimer with the risk of AKI in elderly patients.

| Subgroup |  | Log IBI < median & D-dimer < median | Log IBI < median & D-dimer >= median | | Log IBI >= median & D-dimer < median | | Log IBI >= median & D-dimer >= median | | P for interaction |
| --- | --- | --- | --- | --- | --- | --- | --- | --- | --- |
|  |  |  | OR (95% CI) | P-value | OR (95% CI) | P-value | OR (95% CI) | P-value |  |
| Age | <70 | Reference | 1.669 (1.302-2.138) | <0.001 | 1.965 (1.573-2.458) | <0.001 | 2.559 (2.101-3.130) | <0.001 | 0.925 |
|  | >=70 |  | 1.737 (1.425-2.119) | <0.001 | 1.941 (1.600-2.358) | <0.001 | 2.651 (2.250-3.138) | <0.001 |  |
| Gender | Female |  | 1.401 (1.093-1.796) | 0.008 | 1.957 (1.531-2.503) | <0.001 | 2.312 (1.885-2.852) | <0.001 | 0.153 |
|  | Male |  | 1.958 (1.608-2.385) | <0.001 | 1.986 (1.656-2.387) | <0.001 | 2.838 (2.418-3.343) | <0.001 |  |
| Surgery | No |  | 2.377 (1.853-3.057) | <0.001 | 2.910 (2.276-6.747) | <0.001 | 4.082 (3.295-5.112) | <0.001 | <0.001 |
|  | Yes |  | 1.630 (1.320-2.010) | <0.001 | 1.584 (1.312-1.912) | <0.001 | 2.074 (1.752-2.459) | <0.001 |  |
| Hypertension | No |  | 1.881 (1.525-2.374) | <0.001 | 2.006 (1.635-2.465) | <0.001 | 2.642 (2.219-3.161) | <0.001 | 0.574 |
|  | Yes |  | 1.538 (1.222-1.933) | <0.001 | 1.896 (1.539-2.338) | <0.001 | 2.580 (2.148-3.112) | <0.001 |  |
| Diabetes | No |  | 1.743 (1.466-2.074) | <0.001 | 1.866 (1.577-2.210) | <0.001 | 2.555 (2.214-2.957) | <0.001 | 0.651 |
|  | Yes |  | 1.620 (1.157-2.264) | <0.001 | 2.192 (1.633-2.956) | <0.001 | 2.611 (2.002-3.437) | <0.001 |  |
| Hyperlipidemia | No |  | 1.792 (1.530-2.100) | <0.001 | 1.991 (1.714-2.316) | <0.001 | 2.679 (2.353-3.059) | <0.001 | 0.061 |
|  | Yes |  | 0.645 (2.639-1.416) | 0.300 | 1.601 (0.809-3.083) | 0.166 | 2.386 (1.299-4.402) | 0.005 |  |
| CKD | No |  | 1.767 (1.506-2.074) | <0.001 | 1.971 (1.695-2.294) | <0.001 | 2.693 (2.363-3.076) | <0.001 | 0.521 |
|  | Yes |  | 1.204 (0.670-2.174) | 0.534 | 1.946 (1.104-2.174) | 0.022 | 2.016 (1.244-3.365) | 0.006 |  |
| CAD | No |  | 1.791 (1.486-2.161) | <0.001 | 2.049 (1.714-2.453) | <0.001 | 2.807 (2.409-3.248) | <0.001 | 0.152 |
|  | Yes |  | 1.595 (1.208-2.103) | 0.001 | 1.779 (1.376-2.302) | <0.001 | 2.139 (1.699-2.706) | <0.001 |  |
| Pneumonia | No |  | 1.682 (1.406-2.012) | <0.001 | 1.909 (1.602-2.274) | <0.001 | 2.578 (2.220-3.000) | <0.001 | 0.979 |
|  | Yes |  | 1.795 (1.311-2.476) | <0.001 | 2.005 (1.506-2.700) | <0.001 | 2.746 (2.125-3.605) | <0.001 |  |
| Liver disease | No |  | 1.664 (1.396-1.983) | <0.001 | 1.845 (1.565-2.176) | <0.001 | 2.466 (2.140-2.849) | <0.001 | 0.008 |
|  | Yes |  | 2.114 (1.515-2.980) | <0.001 | 2.529 (1.827-3.538) | <0.001 | 3.340 (2.512-4.519) | <0.001 |  |
| Cerebrovascular disease | No |  | 1.759 (1.486-2.084) | <0.001 | 2.013 (1.716-2.363) | <0.001 | 2.584 (2.248-2.978) | <0.001 | 0.245 |
|  | Yes |  | 1.436 (0.985-2.094) | 0.059 | 1.533 (1.068-2.209) | <0.001 | 2.293 (1.690-3.154) | <0.001 |  |
| Cancer | No |  | 1.796 (1.481-2.179) | <0.001 | 1.922 (1.594-2.319) | <0.001 | 2.495 (2.128-2.935) | <0.001 | 0.963 |
|  | Yes |  | 1.582 (1.223-2.047) | <0.001 | 1.921 (1.518-2.440) | <0.001 | 2.676 (2.172-3.184) | <0.001 |  |

**Abbreviations**: CAD, coronary artery disease; CI, confidence interval; CKD, chronic kidney disease; OR, odds ratio

Table S7. Mediation analysis of D-dimer in Log IBI and the incidence of AKI.

| Effect | Estimate | 95% CI low | 95% CI upp | P-value |
| --- | --- | --- | --- | --- |
| ACME | 0.00145 | 0.00119 | 0.00172 | < 0.001 |
| ADE | 0.00320 | 0.00231 | 0.00412 | < 0.001 |
| Total Effect | 0.00465 | 0.00377 | 0.00554 | < 0.001 |
| Prop. Mediated | 0.31301 | 0.24142 | 0.39700 | < 0.001 |

**Abbreviations**: ACME, average causal mediation effect; ADE, average direct effect; CI, confidence interval

Adjusted for age, gender, smoking status, surgery, hypertension, diabetes, hyperlipidemia, CKD, CAD, CHF, pneumonia, liver disease, cerebrovascular disease, cancer, nephrotoxic antibacterial drugs, contrast agents, chemotherapy drugs, diuretics, NSAIDs, white blood cell count, monocyte count, platelet count, hemoglobin, LDH, uric acid, urea, urine protein, HCO3-, glucose, TC, PT, ALT, and AST.

Table S8. Mediation analysis of Log IBI in D-dimer and the incidence of AKI.

| Effect | Estimate | 95% CI low | 95% CI upp | P-value |
| --- | --- | --- | --- | --- |
| ACME | 0.00026 | 0.00019 | 0.00034 | < 0.001 |
| ADE | 0.00401 | 0.00333 | 0.00474 | < 0.001 |
| Total Effect | 0.00428 | 0.00362 | 0.00498 | < 0.001 |
| Prop. Mediated | 0.06177 | 0.04198 | 0.08550 | < 0.001 |

**Abbreviations**: ACME, average causal mediation effect; ADE, average direct effect; CI, confidence interval

Adjusted for age, gender, smoking status, surgery, hypertension, diabetes, hyperlipidemia, CKD, CAD, CHF, pneumonia, liver disease, cerebrovascular disease, cancer, nephrotoxic antibacterial drugs, contrast agents, chemotherapy drugs, diuretics, NSAIDs, white blood cell count, monocyte count, platelet count, hemoglobin, LDH, uric acid, urea, urine protein, HCO3-, glucose, TC, PT, ALT, and AST.
